# Influence of Zinc Feeding on Nutritional Quality, Oxidative Stability and Volatile Profile of Fresh and Ripened Ewes’ Milk Cheese

**DOI:** 10.3390/foods8120656

**Published:** 2019-12-07

**Authors:** Camillo Martino, Andrea Ianni, Lisa Grotta, Francesco Pomilio, Giuseppe Martino

**Affiliations:** 1 Specialist Diagnostic Department, Istituto Zooprofilattico Sperimentale dell’Abruzzo e del Molise “G. Caporale” Via Campo Boario, 64100 Teramo, Italy; c.martino@izs.it; 2 Department of Medical, Oral and Biotechnological Sciences, “G. d’Annunzio” University of Chieti-Pescara, Via dei Vestini 31, 66100 Chieti, Italy; andreaianni@hotmail.it; 3 Faculty of Bioscience and Technology for Food, Agriculture and Environment, University of Teramo, Via Renato Balzarini 1, 64100 Teramo, Italy; lgrotta@unite.it; 4 Food Hygiene Unit, NRL for L. monocytogenes, Istituto Zooprofilattico Sperimentale dell’Abruzzo e del Molise “G. Caporale” Via Campo Boario, 64100 Teramo, Italy; f.pomilio@izs.it

**Keywords:** zinc, ewes’ milk cheese, rumenic acid, zinc-dependent enzyme, volatile compound

## Abstract

Zinc represents a ubiquitous element in cells with relevant roles in the metabolism of essential nutrients in animals. The aim of this study was to investigate the effect of dietary zinc supplementation on nutritional and aromatic properties of milk and Pecorino cheeses obtained from lactating ewes. Fifty-two commercial ewes were randomly assigned to two groups. The control group was fed with a conventional complete diet, while the experimental group received a daily supplementation of 375 mg/head of zinc oxide. At the end of the trial, which lasted 30 days, samples of milk and related cheese were collected in order to obtain information about the chemical composition and volatile profile. The experimental feeding strategy induced a significant increase in zinc concentration in milk. Furthermore, both in milk and cheese, was observed an increase in vaccenic, rumenic and total polyunsaturated fatty acids, with the consequent significant reduction of atherogenic and thrombogenic indices. The volatile profile of dairy products was also positively affected by dietary zinc intake, with an increase in concentration of hexanoic acid and ethyl esters. The present study suggests interesting possible effects of dietary zinc supplementation of ewes in improving the nutritional characteristics of fresh and ripened dairy products, although more specific and in-depth assessments should be performed on these new products, in order to characterize potential variations on consumers acceptability.

## 1. Introduction

Zinc (Zn) belongs to the family of transition metals and the considerable importance of this microelement lies in its fundamental role in the correct execution of several biochemical mechanisms which mostly provide for the activity of zinc-dependent enzymes [1]. Zn is not stored in animal body, for that reason a constant dietary supply is necessary in order to avoid the onset of a wide range of pathological conditions, such as skin parakeratosis, reduced or cessation of growth, general debility, lethargy and increased susceptibility to infection [2].

It has been reported that almost the half of the soils in the world may be zinc deficient, causing decreased Zn content in plant. In many of these areas, where grazing livestock is widespread, zinc deficiency is prevented by zinc fertilization of pastures. For livestock under more defined conditions, such as poultry, swine, and dairy cattle, feeds are enriched with zinc salts to prevent deficiency [3]. The essentiality of Zn in livestock nutrition is well established, for this reason several feeding strategies have been tested over time to ensure the adequate dietary intake of all the necessary trace elements. In addition to this, the concentration of these elements in milk and dairy products has been reported to be heavily influenced by the feeding strategy. Regarding Zn, the chemical form mostly used for the industrial preparation of animal feeds is represented by zinc oxide (ZnO), although it has recently been introduced the nano zinc oxide (nZnO) in an attempt to improve solubility and Zn availability, without inducing toxicity [4].

Nutritional requirements of ruminants are different from those of monogastric animals. Several studies showed that the bioavailability of specific trace elements is of primary relevance in supporting an adequate ruminal fermentation and digestion. Sonawane and Arora [5] conducted an in vitro study in which observed an increased synthesis of microbial proteins as a consequence of ruminal fluid incubation with additional Zn as ZnCl_2_ or ZnSO_4_; more recently, has been demonstrated the Zn ability to inhibit the ruminal hydrolysis of urea when fed to steers consuming low quality hay, therefore avoiding the excessive increase in NH_3_ concentration which could negatively interfere with protein synthesis by ruminal microbes [6]. In ewes, the extra dietary Zn supplementation was also reported to induce the transcriptional modulation of protein mediators of cellular signaling, cardiac contractility and immune response [7].

Over time different studies have been performed with the aim to evaluate productive and qualitative parameters of milk obtained from ruminants fed a dietary supplementation of organic and inorganic Zn. Salama et al. [8] reported that milk yield was not significantly affected by Zn-methionine intake in dairy goats [8], furthermore no variations were observed concerning the percentages of protein, lactose, fat, solid non-fat, total solid, and density of milk. Recently, Ianni et al. [9] confirmed this finding in lactating dairy cows supplemented with ZnO, also highlighting an improvement in the nutraceutical properties of milk, due to the increased concentration of conjugated linoleic acids (CLA). In similar studies has been also found an increase in Zn concentration both in milk and in bovine cheese, as evidence of the fact that changes in animal feeding represent promising approaches to modify Zn amount in milk and related dairy products [10,11].

The objective of this study was to assess the influence of a dietary zinc oxide supplementation in lactating ewes on nutritional characteristics, fatty acids composition, lipid peroxidation and volatile profile of fresh and 90-days ripened ewes’ milk cheese. There are adequate evidences that would support a positive role for Zn in influencing the biochemical mechanisms directly involved in defining the qualitative parameters of the animal production.

## 2. Materials and Methods

### 2.1. Experimental Design, Cheese Manufacturing Protocol and Sampling

Fifty-two half-bred ewes have been randomly divided into two groups: a control group (CG) and an experimental group (EG) whose diet was supplemented with Zn. Individual milk samples were collected before the trials to obtain information about milk yield, chemical composition and fatty acid profile. This approach was useful to verify the eventual presence of variations among the selected groups.

For 30 days, the CG received a complete diet that was prepared in accordance with the sheep nutritional needs, and guaranteeing each animal the daily Zn requirement of about 79 mg. The EG received the same complete food, formulated according to the same requirements and prepared in the same way, however enriching the daily ration of each sheep with additional 296 mg Zn in order to obtain a total intake of about 375 mg. The management of Zn doses was executed according to the Regulation (EC) No. 1831/2003 of the European Parliament and of the Council of 22 September 2003 on additives for use in animal nutrition [12].

With regard to the Pecorino cheese, the production was performed by the same company in which the trial was conducted, located in the province of Teramo (Abruzzo, Italy). The manufacturing protocol provided the bulk ewes’ milk pasteurization at 70 ± 1 °C for 15 s, followed by cooling at 40 °C and inoculation with a freeze-dried starter culture (*Streptococcus thermophilus*, *Lactobacillus casei* and *Lactobacillus delbrueckii* subsp. *bulgaricus*) produced by FERM IN (ChemiFerm s.r.l., Livraga, Italy). Then, milk was coagulated by adding kid rennet paste (75% chymosin and 25% pepsin; 1:18,000 strength; Clerici, Cadorago, Italy) and the curd was subsequently cut into small pieces by stirring with a spatula, heated to 42 ± 1 °C and manually pressed. The resulting cheeses, of about 1 kg each, were held at 10 °C until the next day, when they were salted in aqueous solution containing 18% of sodium chloride for 12 h. Ripening was conducted at 12 ± 1 °C.

With the purpose of evaluating variations in chemical composition and quality attributes due to ripening, the sampling of ewes’ milk cheese was carried out after 1 (T_1_) and 90 (T_90_) days from the cheese-making. Samples, collected in triplicate from three different cheese-makings, were partly immediately analyzed and partly packed under vacuum and frozen at −20 °C until analysis.

### 2.2. Chemical Analysis of Milk and Cheese

MilkoScan FT 6000 (Foss Integrator IMT; Foss, Hillerød, Denmark) was used to determine the chemical composition of milk (fat, protein, casein, lactose, and urea), while somatic cells count (SCC) and total bacterial count (TBC) were performed using respectively the Fossomatic TM FC and the BactoScan FC (Foss, Hillerød, Denmark). In cheese, the evaluation of pH, moisture, total proteins, lipids and ash were performed according to AOAC methods (1990) [13]; water-soluble nitrogen (WSN) and trichloroacetic acid-soluble nitrogen (TCA-SN) were determined according to the International Standard ISO 27871 IDF 224 (2011) [14], and results have been reported as percentage of total nitrogen, following appropriate calibration.

The total amount of Zn in milk and cheese was determined by inductively coupled plasma mass spectrometry (ICP-MS) by using an Agilent 7500ce (Agilent Technologies, Palo Alto, CA, USA) and following the procedure reported by Gerber et al. [15] with slight modifications. Samples, 5 mL of milk or 5 g of cheese, were accurately inserted into quartz digestion vessels. At this point, 3 mL of 30 % hydrogen peroxide (Sigma Aldrich, Milan, Italy) and 10 mL 65 % nitric acid (Sigma Aldrich, Milan, Italy) were added to each tube, which was then closed for sample digestion at 95 °C for 2 h. After the vessels had cooled down, the digests were transferred into 50 mL volumetric flasks and filled to the mark using ultrapure water. One milliliter of the solution was added with 9 mL of distilled nitric acid (1%) and analyzed. The Zn determination was performed by referring to a calibration and results were expressed in mg/kg.

### 2.3. Evaluation of Fatty Acid Profile in Milk and Cheese

Extraction of the milk lipid fraction was made according to the AOAC official method [16], while in cheese was used a mix of chloroform and methanol (2:1, *v*/*v*; Sigma Aldrich, Milan, Italy). Trans-methylation of lipid extracts and separation of fatty acyl methyl esters (FAMEs) was performed according to the procedure reported by Ianni et al. [17]. Individual FAMEs were identified by comparing the retention time of a standard mixture (FIM-FAME7-Mix; Matreya LLC, Pleasant Gap, PA, USA), and individual C18:1 *trans*-11 and C18:2 *cis*-9, *trans*-11 (Matreya LLC). The ChromeCard software was used for the quantification of peak areas, and each FAME was expressed as a percentage of the total FA. These values were used to obtain the sum of saturated (SFA), monounsaturated (MUFA) and polyunsaturated fatty acids (PUFA). Furthermore, atherogenic and thrombogenic indices (AI and TI, respectively) were calculated in milk and ewes’ cheese using the formulas proposed by Ulbricht and Southgate [18], whereas the desaturation index (DI) was defined as proposed by Mele et al. [19].

### 2.4. Evaluation of Lipid Peroxidation by TBARS-Test

Lipid peroxidation in Pecorino cheese was determined by evaluating the amount of thiobarbituric acid reactive substances (TBARS). The analysis was performed in accordance with the procedure described by Bennato et al. [20] with slight modifications. Five grams of frozen cheese were mixed, within 2 min of sample withdrawal from the freezer, with 500 µL of 0.1% of butylated hydroxytoluene (BHT; Sigma Aldrich, Milan, Italy) in methanol to block the oxidation process. The mixture was homogenized with Ultra Turrax T-25 high speed homogenizer (IKA, Staufen, Germany) in 50 mL of an aqueous solution containing 7% trichloroacetic acid (TCA; Sigma Aldrich, Milan, Italy), and then distilled (ASTORI Tecnica s.n.c., Poncarale, BS, Italy). For each distillate, 2 mL were mixed with an equal volume of a 0.02 M thiobarbituric acid (TBA; Sigma Aldrich, Milan, Italy) solution in 90% acetic acid and then heated up to 80 °C in a thermostated bath, keeping the temperature constant for 1 hour. The absorbance at 534 nm was evaluated with a spectrophotometer (Jenway, Essex, UK) after cooling. The malondialdehyde (MDA) amount in each sample was calculated by referring to a calibration curve ranging from 0 to 100 ppm (*R*^2^ = 0.989), and results were expressed in µg of MDA per g of cheese.

### 2.5. Analysis of Volatile Compounds

Volatile compounds (VOC) were extracted from Pecorino cheese samples through solid-phase microextraction (SPME), and the analysis was performed with a gas chromatograph (Clarus 580; Perkin Elmer, Waltham, MA, USA) coupled with a mass spectrometer (SQ8S; Perkin Elmer, Waltham, MA, USA). The gas chromatograph was equipped with an Elite-5MS column (length × internal diameter: 30 × 0.25 mm; film thickness: 0.25 μm; Perkin Elmer, Waltham, MA, USA). The samples preparation and the settings relating to the thermal program used for the analysis were performed as previously reported by Ianni et al. [21]. Five grams of cheese previously grated were mixed with 10 mL of saturated NaCl solution (360 g/L). After the addition of 10 μL of internal standard solution (4-methyl-2-heptanone; 10 mg/kg in ethanol), the vials were sealed and stirred at 50 °C; VOCs were extracted from the headspace with a divinylbenzene-carboxen-polydimethylsiloxane SPME fiber (length: 1 cm; film thickness: 50/30 μm; Supelco, Bellefonte, PA, USA) with an exposition time of 60 min. VOCs were identified by comparison with mass spectra of a library database (NIST Mass Spectral library, Search Program version 2.0, National Institute of Standards and Technology, U.S. Department of Commerce, Gaithersburg, MD, USA) and by comparing the eluting order with Kovats indices.

### 2.6. Statistical Analysis

All analyses were performed at least in triplicate and results were reported as mean ± standard deviation. The SigmaPlot 12.0 software (Systat software, Inc., San Jose, CA, USA) for Windows operating system was used to analyze the statistical significance of the differences between the averages for each group (ANOVA, Student’s *t*-test); *p* values lower than 0.05 were considered statistically significant.

## 3. Results

### 3.1. Chemical Composition of Milk and Cheese

Taking into account the milk production over the entire duration of the dietary zinc enrichment, no significant differences were evidenced between the two groups, reflecting the fact that such parameter was not affected by diet. Regarding the chemical quality of milk (Table 1), all the analyzed parameters did not undergo variations during the experimental period. Similarly, no significant differences were observed as regards the ureic content and pH, whereas the EG samples showed a lower SCC with respect to the CG (*p* < 0.01). In regards to the amount of Zn, in the experimental group were found higher average values (4.82 ± 0.23 vs 5.42 ± 0.34 mg/kg, in CG and EG respectively; *p* < 0.05).

As evidenced for milk, the dietary supplementation did not influence cheese yield (*p* > 0.05). Regardless of the feeding strategy, no significant differences in composition of cheeses were evidenced (Table 2). Regarding the ripening time, as expected a significant reduction in moisture was found in T_90_ samples (*p* < 0.05); protein and lipids were not influenced by ripening, as well as the zinc amount which maintained similar values between the two groups. Furthermore, in T_90_ samples obtained from EG, the nitrogen fractions were significantly higher (0.94% vs. 0.63%, *p* < 0.05, for WSN; 0.56% vs. 0.35%, *p* < 0.05, for TCA-SN).

### 3.2. Fatty acid Profile of Milk and Cheese

The fatty acid composition of individual milk samples collected at the beginning and at the end of the trial by both the experimental groups is reported in Table 3. At T_0_ not significant variations between CG and EG were evidenced testifying to the homogeneity of the animals selected for the study. At the end of the experimental period, samples of milk obtained from EG evidenced an increase in the content of vaccenic acid (C18:1 *trans*11; *p* < 0.05), rumenic acid (RA; *p* < 0.01) and total polyunsaturated fatty acids (PUFA, *p* < 0.05).

Similarly, the evaluation of the total fatty acids profile in cheese evidenced modifications already evident in milk (Table 3), with an increase in concentration, in EG, of vaccenic acid (C18:1 trans11; *p* < 0.01), rumenic acid (RA; *p* < 0.05) and total PUFA (*p* < 0.05). Based on the obtained FA profile, calculations of desaturation, thrombogenic and atherogenic indices were performed. Dietary supplementation with zinc did not induce significant modifications of desaturation index (*p* > 0.05) both in milk and cheese; thrombogenic index was significantly lower only in cheese samples obtained from EG (*p* < 0.05), whereas atherogenic index decreased both in milk and cheese obtained from the EG (*p* < 0.05).

### 3.3. Analysis of the Oxidative Stability in Pecorino Cheese

**Figure 1:**
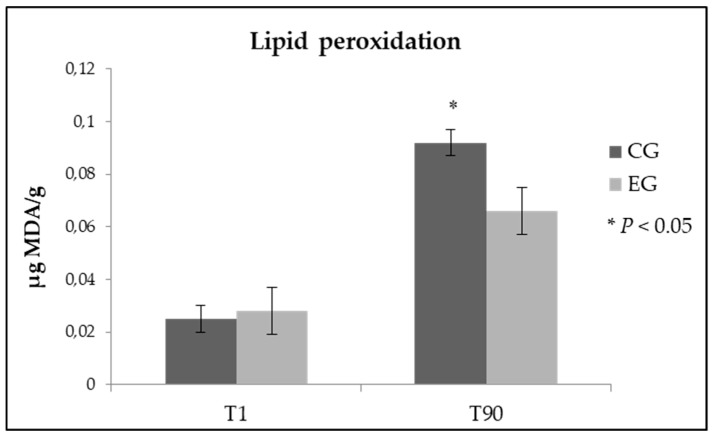
Lipid peroxidation in fresh and ripened Pecorino cheese samples obtained from control group (CG) and experimental group (EG).

Diet enrichment with zinc did not induce alterations of the oxidative stability in samples of fresh cheese (Figure 1). Very interesting is instead the result obtained after 90 days from the cheese making, as expected lipid peroxidation increased in all the analyzed samples, but in ewes’ milk cheese obtained from EG the value of malondialdehyde was stood at significantly lower values if compared to samples of the control group (0.092 vs 0.066 µg MDA/g of cheese, in CG and EG respectively; *p* < 0.05).

### 3.4. Volatile Profile of Cheese

The analysis of the volatile profile allowed to identify 24 volatile compounds (VOC) in samples of T_1_ and T_90_ cheese obtained from CG and EG: 6 carboxylic acids, 5 ethyl esters, 3 aldehydes, 2 alcohols, 2 lactones, 2 ketones and 4 classified as aromatic hydrocarbons (Table 4).

Regarding the carboxylic acids, is interesting the increase in concentration of the hexanoic acid in the EG samples after 90 days of ripening (6.75% vs. 2.06% in EG and CG samples respectively; *p* < 0.01); in T_90_ samples should also be underlined the significant reduction of the longer chain acids: decanoic acid (9.67% vs. 5.09% in CG and EG respectively; *p* < 0.01) and dodecanoic acid (9.57% vs. 6.67% in CG and EG respectively; *p* < 0.01). 

In the case of esters, the dietary zinc supplementation induced the increase of butanoic acid ethyl ester (*p* < 0.01) and of hexanoic acid ethyl ester (*p* < 0.05) at the end of the ripening period. Different resulted the behavior of octanoic acid ethyl ester, which is higher in the T_90_ samples obtained from CG (11.85% vs. 7.31%, *p* < 0.01).

In the most ripened samples was also found the hexanal increase in EG samples (9.86% vs. 7.46%, *p* < 0.05), and variations of both nonanal (1.68% vs. 1.12% in CG and EG respectively; *p* < 0.05) and 3-methylbunanol (5.98% vs. 3.27% in CG and EG respectively; *p* < 0.05).

Additionally, in T_1_ samples have been evidenced some significant differences, specifically in EG cheeses have been observed lower concentrations of nonanoic acid (*p* < 0.05), nonanal (*p* < 0.05) and 2-heptanone (*p* < 0.01).

## 4. Discussion

In the present study, Zn enrichment of ewes’ diet did not induce significant changes on milk composition, and this finding is consistent with what has been previously reported in dairy cows [22,23] and dairy goats [8]. According to what was observed in milk, no variations were evidenced in the chemical composition of Pecorino cheese samples, both in relation to dietary treatment and ripening time. The analysis on milk showed instead the capacity of the experimental diet to markedly reduce the number of somatic cells, a datum already observed in dairy cows by Pechová et al. [24] who tested the effect of dietary Zn at a daily dose of 440 mg/animal, therefore by administering a Zn dose similar to that used in the present work but on animals with a much greater body weight. Such phenomenon was justified by assuming an increased Zn supply into the mammary gland with a consequent improvement of the immune response. A precise and exhaustive evaluation of the influence of this parameter on the quality of dairy products is not feasible, however, it is known that somatic cells contribute to proteolysis in milk and cheese because of their tendency to release proteolytic enzymes in the extracellular environment [25].

A finding deserving special attention concerns the Zn content, which is higher in EG milk samples, whereas no variations were highlighted in cheese. The Zn concentration in cheese could be directly related to the presence of caseins, with which the microelement interacts through a complex kinetic that develops in two phases: an initial rapid stage in which about the 70% of Zn interacts with polar amino acids of casein, and a significantly slower second stage in which the equilibrium is reached [26]. Since after the cheese-making both CG cheese and EG cheese showed similar protein concentrations, it could be supposed that in EG samples the Zn excess, not associated with caseins, may have been lost with serum after rennet breakage. In addition to this, it should be also noted that in this study Zn seems to show a moderate capacity for association with caseins, as can be proved by the reduced Zn concentration in cheeses as compared with milk (considering the casein concentration by cheesemaking).

The Zn enrichment of ewes’ diet showed effective in inducing variations in the FA profile, both in milk and its derived cheese; in all EG samples, the amount of vaccenic acid (C18:1 *trans*11), rumenic acid (RA) and total PUFA significantly increased. With regard to vaccenic acid, the result is explainable, at least in part, by taking into account the study of Szczechowiak et al. [27], in which the increase in concentration of this compound was justified by the action of bioactive compounds taken through the diet which tend to slow down or totally inhibit the terminal steps of ruminal biohydrogenation, thus avoiding the formation of stearic acid (C18:0). The relevance of vaccenic acid is its role as substrate of the mammary gland stearoyl coenzyme A desaturase (SCD), an endoplasmic reticulum-bound enzyme which is responsible for the catalytic mechanism that gives origin to CLA [28,29,30]. In this study the RA concentration effectively increased in both milk and cheese obtained from the experimental group. A similar finding was recently reported by Ianni et al. [9] who tested a dietary zinc supplementation in lactating dairy cows. In that case, the phenomenon was partly justified by advancing the hypothesis of a role of the Zn supplemented diet in promoting the catalytic function of site-2 protease (S2P), a Zn-dependent metalloprotease which contributes to the activation of the sterol response element binding protein (SREBP), a transcription factor responsible for the regulation of several genes encoding for SCD and other lipogenic factors.

RA has been indicated as a factor with a strong antioxidant function which is able to improve the mammary gland functionality by protecting the mammary epithelial cells from lipoperoxidation through the reduction of the reactive oxygen species [31]. For humans, the ruminant products represent the primary dietary source of CLA, which are credited of several health benefits; the most relevant examples concern the modulation of the immune system response [32], and their potential activity in slowing down the progression of different pathological conditions, as in the case of atherosclerosis [33].

Both in milk and its derived cheese, the feeding strategy based on Zn enrichment also induced an overall increase of PUFA concentration, at the expense of SFA. As a direct consequence of this evidence, the atherogenic and thrombogenic indices decreased, testifying a noteworthy improvement of the health functionality of animal productions.

The oxidative damage in cheese was determined by evaluating the amount of thiobarbituric acid reactive substances. Zn has been reported to act as a free radical scavenger in biological systems, with the consequent inhibition of free radical lipid peroxidation [34]. Additionally, in this study, Zn appeared to provide antioxidant protection since at the end of the 90 days of ripening (T_90_) the TBA values, although rising in both experimental groups, were found to be significantly lower in cheese samples deriving from the dietary Zn supplementation.

The analysis performed with the purpose to characterize the volatile profile of fresh (T_1_) and ripened (T_90_) ewes’ milk cheese, led to the identification of different families of compounds, the most represented of which are those of free fatty acid (FFA) and ethyl esters. The biochemical mechanism behind the increased concentration of FFA could relate to the degradation of triglycerides by enzymes of both endogenous and microbial origin. Among FFA the hexanoic acid was present at higher concentrations in EG samples after 90 days of ripening. This compound can only originate from lipolysis [35], and the datum concerning its increase in concentration, should have a significant influence in the determination of cheese flavor, due to its association with strong odors, described as cheesy, rancid and sweaty. In addition to this, the increase of hexanoic acid could also derive from the lipolytic action on longer chain acids, decanoic and dodecanoic, which in fact undergo a marked reduction in the EG samples [36]. What has just been described would suggest a condition characterized by an increase in lipolysis during the ripening period. Some authors explain this phenomenon with an increase in the autolysis of bacterial starters, with the consequent release in the extracellular environment of a wide range of enzymes capable to attack and degrade both the protein component and fatty acids, contributing to the development of the organoleptic properties in ripened cheeses [37]. The autolysis process is, in turn, mediated by peptidoglycan hydrolases, named autolysins, that in the presence of certain stimuli degrade the cellular envelope which separates the cytoplasmic compartment from the extracellular matrix [38,39].

The FFAs, in addition, to directly contribute to the cheese flavor, represent the substrate for the biosynthesis of other classes of compounds: ethyl esters, methyl ketones, secondary alcohols, aldehydes, and lactones [36]. With regard to esters, the EG samples analyzed at the end of the ripening period were characterized by higher concentrations of butanoic acid ethyl ester and hexanoic acid ethyl ester. Generally, this class of compounds is responsible for the supply of sweet, fruity, and floral notes in surface ripened cheese [40], and is considered to be decisive in defining the cheese flavor because of the low odor threshold. Among aldehydes, was instead evidenced a marked increase of hexanal after ripening in EG samples; this compound gives what is called "green grass-like” aroma, with lemon, herbal and slightly fruity notes. A strange behavior is associated to alcohols which mainly derive from FFAs catabolism; after the 90 days of ripening, these compounds are not well represented, and their concentration tends to decrease in the EG samples. Specifically, we found a significant reduction of 3-methylbutanol, which is generally found on surface-ripened cheese and is responsible for alcoholic, fruity, grainy, and solvent-like notes [34]. No variations were evidenced for lactones and ketones, therefore further analysis could be necessary in order to better characterize the biochemical mechanisms that contribute to their biosynthesis.

## 5. Conclusions

The experimented feeding strategy showed able to modify the nutritional properties of ewes’ milk and its derived Pecorino cheese. The main finding concerns the increase in concentration of vaccenic acid, rumenic acid and total PUFA both in milk and cheese, as evidence of a presumable improvement in the health functionality of the products. In addition to this, a clear improvement of the oxidative stability was also shown in the 90-days aged cheese, an aspect that deserves interesting implications for the preservation of the cheese quality during ripening. The volatile profile of ewes’ milk cheese was also positively affected by the dietary Zn intake. Of particular interest, with a view to improving the cheese aroma, is the increase in concentration in hexanal and 3-methylbutanol at the end of ripening. However, it would be necessary to plan a specific investigation aimed at assessing the approval rate of these new products by consumers.

## Figures and Tables

**Table 1:** Milk yield and chemical composition of milk obtained from the control group (CG) and the experimental group (EG).

	Diet		*p*
	CG	EG	
Animal Parameters			
Milk yield (mL/day)	778 ± 38	810 ± 45	n.s.
Item			
Fat (%)	7.53 ± 0.58	7.49 ± 0.41	n.s.
Protein (%)	6.03 ± 0.48	6.17 ± 0.45	n.s.
Casein (%)	4.78 ± 0.37	4.89 ± 0.29	n.s.
Lactose (%)	4.64 ± 31	4.63 ± 0.26	n.s.
Urea (mg/100 mL)	57.68 ± 4.21	57.72 ± 3.74	n.s.
SCC (LS) ^1^	5.51 ± 0.12	5.07 ± 0.09	**
Total bacterial count (TBC, UFC/mL 103)	686 ± 72	633 ± 54	n.s.
pH	6.54 ± 0.13	6.55 ± 0.09	n.s.
Zinc (mg/kg)	4.82 ± 0.23	5.42 ± 0.34	*

Data are expressed as mean ± S.D. ^1^ Somatic cell count (SCC) is reported in linear score (LS): LS = log_2_[(cells/µL)/100] + 3. *p < 0.05; ** *p* < 0.01; n.s. = not significant.

**Table 2:** Chemical composition of Pecorino cheese obtained from the control group (CG) and the experimental group (EG), analyzed after 1 (T_1_) and 90 (T_90_) days after the cheese-making.

	T_1_		T_90_	
Item	CG	EG	CG	EG
Moisture (%)	46.35 ^A^ ± 2.12	49.47 ^A^ ± 2.23	37.66 ^B^ ± 1.54	39.31 ^B^ ± 1.68
Fat ^1^ (%)	28.44 ± 2.03	27.86 ± 1.87	25.08 ± 1.93	23.98 ± 1.79
Protein^1^ (%)	22.03 ± 2.14	19.89 ± 1.54	21.18 ± 1.64	19.04 ± 1.65
WSN (%N)	0.58 ± 0.06	0.63^A^ ± 0.07	0.64 ^a^ ± 0.06	0.94 ^b,B^ ± 0.08
12% TCA-SN (%N)	0.27 ± 0.03	0.35^A^ ± 0.04	0.33 ^a^ ± 0.04	0.56 ^b,B^ ± 0.05
pH	6.41 ± 0.15	6.64 ± 0.17	5.86 ^a^ ± 0.15	5.46 ^b^ ± 0.15
Zinc (mg/kg)	2.21 ± 0.18	2.16 ± 0.19	1.96 ± 0.21	1.88 ± 0.20

Data are expressed as mean percentage ± S.D. ^1^ Data are reported on a dry matter (DM) basis. ^a,b^ Means with different superscripts are significantly different by diet (*p* < 0.05). ^A,B^ Means with different superscripts are significantly different by ripening time (*p* < 0.01).

**Table 3:** Fatty acid profile of milk and fresh cheese obtained from the control group (CG) and the experimental group (EG).

	Milk						Fresh Cheese		
	T_0_			T_30_					
	CG	EG	*p*	CG	EG	*p*	CG	EG	*p*
C4:0	2.21 ± 0.19	2.03 ± 0.18	n.s.	2.02 ± 0.17	1.98 ± 0.16	n.s.	2.16 ± 0.18	2.08 ± 0.16	n.s.
C6:0	2.14 ± 0.18	2.18 ± 0.16	n.s.	2.06 ± 0.16	1.94 ± 0.16	n.s.	1.98 ± 0.16	1.89 ± 0.17	n.s.
C8:0	2.23 ± 0.20	1.91 ± 0.16	n.s.	2.28 ± 0.17	2.16 ± 0.18	n.s.	2.33 ± 0.19	2.25 ± 0.20	n.s.
C10:0	7.45 ± 0.61	7.33 ± 0.56	n.s.	7.89 ± 0.54	7.50 ± 0.61	n.s.	7.76 ± 0.64	7.41 ± 0.59	n.s.
C11:0	0.27 ± 0.03	0.33 ± 0.03	n.s.	0.31 ± 0.03	0.29 ± 0.03	n.s.	0.35 ± 0.03	0.32 ± 0.03	n.s.
C12:0	5.16 ± 0.49	4.61 ± 0.42	n.s.	5.00 ± 0.36	4.49 ± 0.34	n.s.	5.21 ± 0.44	4.71 ± 0.41	n.s.
C14:0	13.08 ± 1.12	12.11 ± 0.97	n.s.	12.99 ± 1.03	11.76 ± 0.98	n.s.	13.26 ± 0.99	12.02 ± 1.02	n.s.
C15:0	1.29 ± 0.11	1.22 ± 0.12	n.s.	1.20 ± 0.15	1.18 ± 0.09	n.s.	1.16 ± 0.12	1.11 ± 0.11	n.s.
C16:0	25.12 ± 2.14	26.53 ± 2.09	n.s.	26.28 ± 1.83	27.00 ± 1.65	n.s.	26.58 ± 2.07	27.09 ± 2.24	n.s.
C17:0	0.61 ± 0.06	0.52 ± 0.05	n.s.	0.53 ± 0.04	0.49 ± 0.04	n.s.	0.55 ± 0.05	0.43 ± 0.04	n.s.
C18:0	6.78 ± 0.62	7.04 ± 0.68	n.s.	7.97 ± 0.61	7.29 ± 0.63	n.s.	7.59 ± 0.65	6.88 ± 0.58	n.s.
SFA	66.34 ± 3.67	65.81 ± 3.11	n.s.	68.53 ± 2.98	66.08 ± 3.02	n.s.	68.93 ± 3.05	66.19 ± 2.27	n.s.
C14:1	0.85 ± 0.08	0.88 ± 0.08	n.s.	0.82 ± 0.07	0.86 ± 0.09	n.s.	0.83 ± 0.06	0.89 ± 0.08	n.s.
C16:1	1.02 ± 0.09	0.95 ± 0.09	n.s.	0.96 ± 0.07	1.12 ± 0.09	n.s.	1.15 ± 0.09	1.21 ± 0.10	n.s.
C18:1 *trans*11	0.72 ± 0.07	0.75 ^a^ ± 0.05	n.s.	0.67 ± 0.06	0.88 ^b^ ± 0.07	*	0.57 ± 0.06	0.79 ± 0.07	**
C18:1 *cis*9	18.13 ± 1.24	17.89 ± 1.31	n.s.	17.40 ± 1.12	17.94 ± 1.19	n.s.	17.73 ± 1.24	19.85 ± 1.41	n.s.
C18:1 *cis*11	0.55 ± 0.06	0.71 ± 0.07	n.s.	0.51 ± 0.06	0.62 ± 0.06	n.s.	0.44 ± 0.04	0.57 ± 0.06	n.s.
MUFA	21.27 ± 1.78	21.18 ± 1.23	n.s.	20.36 ± 1.17	21.42 ± 1.24	n.s.	20.72 ± 1.21	23.31 ± 2.13	n.s.
C18:2	1.78 ± 0.14	1.85 ± 0.16	n.s.	1.84 ± 0.13	2.09 ± 0.16	n.s.	1.66 ± 0.14	1.71 ± 0.16	n.s.
C18:3	1.01 ± 0.09	0.92 ± 0.09	n.s.	0.86 ± 0.07	0.96 ± 0.08	n.s.	0.75 ± 0.07	0.83 ± 0.08	n.s.
RA	1.71 ± 0.11	1.67 ^a^ ± 0.13	n.s.	1.73 ± 0.14	2.13 ^b^ ± 0.16	**	1.56 ± 0.15	1.89 ± 0.17	*
C20:4	0.05 ± 0.00	0.06 ± 0.00	n.s.	0.07 ± 0.01	0.06 ± 0.01	n.s.	0.04 ± 0.00	0.04 ± 0.01	n.s.
PUFA	4.55 ± 0.38	4.50 ^a^ ± 0.31	n.s.	4.50 ± 0.32	5.24 ^b^ ± 0.39	*	4.01 ± 0.19	4.47 ± 0.26	*
Others	7.84 ± 0.67	8.34 ± 0.71	n.s.	7.71 ± 0.44	7.36 ± 0.53	n.s.	6.44 ± 0.62	5.03 ± 0.48	*
DI	0.06 ± 0.00	0.07 ± 0.01	n.s.	0.06 ± 0.00	0.07 ± 0.01	n.s.	0.06 ± 0.00	0.07 ± 0.01	n.s.
AI	3.20 ± 0.27	3.10 ± 0.16	n.s.	3.35 ± 0.21	2.94 ± 0.16	*	3.43 ± 0.31	2.77 ± 0.25	*
TI	2.97 ± 0.21	3.09 ± 0.24	n.s.	3.33 ± 0.27	3.05 ± 0.25	n.s.	3.41 ± 0.31	2.88 ± 0.26	*

Analysis on milk have been performed at the beginning (T_0_) and at the end of the trial (T_30_). SFA = saturated fatty acid; MUFA = monounsaturated fatty acid; PUFA = polyunsaturated fatty acid; RA = rumenic acid; AI = atherogenic index; TI = thrombogenic index; DI = desaturation index. Data are expressed as mean (%) ± S.D. * p < 0.05; ** *p* < 0.01; n.s. = not significant. ^a,b^ Means with different superscripts (in milk) are significantly different by time (*p* < 0.05).

**Table 4:** Volatile compounds (VOCs) detected in cheese samples obtained from control group (CG) and experimental group (EG).

		T_1_			T_90_		
	VOC	CG	EG	*p*	CG	EG	*p*
**Acids**	acetic acid	1.98 ± 0.14	1.76 ± 0.11	n.s.	2.29 ± 0.17	2.48 ± 0.19	n.s.
	hexanoic acid	2.12 ± 0.17	1.99 ± 0.16	n.s.	2.06 ± 0.18	6.75 ± 0.45	**
	octanoic acid	3.83 ± 0.28	3.56 ± 0.29	n.s.	7.40 ± 0.45	7.74 ± 0.56	n.s.
	nonanoic acid	1.76 ± 0.14	1.34 ± 0.10	*	1.09 ± 0.08	1.24 ± 0.10	n.s.
	decanoic acid	9.63 ± 0.73	10.23 ± 0.88	n.s.	9.67 ± 0.62	5.09 ± 0.39	**
	dodecanoic acid	13.63 ± 1.04	15.23 ± 1.29	n.s.	9.57 ± 0.63	6.67 ± 0.44	**
**Aldehydes**	hexanal	4.11 ± 0.22	4.73 ± 0.37	n.s.	7.46 ± 0.53	9.86 ± 0.24	*
	heptanal	6.88 ± 0.43	8.07 ± 0.67	n.s.	n.d.	n.d.	n.s.
	nonanal	13.29 ± 1.15	10.21 ± 0.77	*	1.68 ± 0.14	1.12 ± 0.09	*
**Alcohols**	3-methylbutanol	4.87 ± 0.29	5.31 ± 0.34	n.s.	5.98 ± 0.36	3.27 ± 0.22	*
	2,3-butanediol	n.d.	n.d.	n.s.	2.72 ± 0.23	2.15 ± 0.18	n.s.
**Esters**	butanoic acid, ethyl ester	n.d.	n.d.	n.s.	4.21 ± 0.31	9.29 ± 0.76	**
	hexanoic acid, ethyl ester	6.57 ± 0.41	6.79 ± 0.42	n.s.	8.96 ± 0.64	11.66 ± 0.98	*
	octanoic acid, ethyl ester	8.32 ± 0.52	7.67 ± 0.39	n.s.	11.85 ± 0.78	7.31 ± 0.56	**
	decanoic acid, ethyl ester	n.d.	n.d.	n.s.	4.10 ± 0.29	4.78 ± 0.38	n.s.
	dodecanoic acid, ethyl ester	n.d.	n.d.	n.s.	0.40 ± 0.05	0.31 ± 0.04	n.s.
**Lactones**	δ-decalactone	3.89 ± 0.26	3.83 ± 0.29	n.s.	1.22 ± 0.10	1.09 ± 0.07	n.s.
	δ-dodecalactone	4.44 ± 0.32	3.86 ± 0.34	n.s.	0.98 ± 0.08	0.85 ± 0.07	n.s.
**Ketones**	2-heptanone	1.91 ± 0.09	1.39 ± 0.08	**	3.18 ± 0.25	2.95 ± 0.22	n.s.
	2-nonanone	n.d.	n.d.	n.s.	0.82 ± 0.06	1.03 ± 0.09	n.s.
**Aromatic hydrocarbons**	ethylbenzene	n.d.	n.d.	n.s.	2.22 ± 0.19	1.94 ± 0.15	n.s.
	1,3-dimethylbenzene	n.d.	n.d.	n.s.	0.27 ± 0.03	0.32 ± 0.03	n.s.
	1,2,3,4-tetramethylbenzene	n.d.	n.d.	n.s.	0.84 ± 0.06	0.95 ± 0.08	n.s.
	*p*-cymene	n.d.	n.d.	n.s.	0.62 ± 0.06	0.77 ± 0.07	n.s.

Data are expressed as mean (%) ± S.D. * *p* < 0.05; ** *p* < 0.01; n.s.: not significant; n.d.: not detectable.
